# A Two-way Mendelian randomisation study of inflammatory factors and the risk of meningioma

**DOI:** 10.1515/tnsci-2025-0389

**Published:** 2026-01-14

**Authors:** Jiming Sun, Xinlei Yang, Han Gao, Rui Lin, Xiaobo Sun, Qiutao Li, Xinyu Chang, Shengxin Bao, Yu Fan, Yiran Du

**Affiliations:** Department of Functional Neurosurgery, Shanghai Eber Hospital, Shanghai, China; Tumor Treatment Center, Affiliated Hospital of Beihua University, Jilin, China; Department of General Surgery, Qian Gorlos Mongolian Autonomous County Hospital, Jilin, China; Outpatient Department, Affiliated Hospital of Beihua University, Jilin, China; Tonghua City Hospital of Chinese Medicine, Jilin, China; Pain Management Department, Qian Gorlos Mongolian Autonomous County Hospital, Jilin, China; Department of Neurovascular Surgery Group3, Jilin Central Hospital, Jilin, China

**Keywords:** inflammatory factors, meningioma, Mendelian randomisation, Fms-related tyrosine kinase 3

## Abstract

**Objectives:**

To explore the causal relationship between inflammatory factors and meningioma.

**Methods:**

The inverse variance weighting method (IVW), Mendelian Randomisation Egger (MR-Egger) regression, weighted median method, simple mode method, and weighted mode method were used to analyse the potential causal relationship between exposure factors and outcomes.

**Results:**

Preliminary MR analysis showed that 6 inflammatory factors, including C-C motif chemokine 19 levels, osteoprotegerin levels, Fms-related tyrosine kinase 3 (FLT3) ligand levels, matrix metalloproteinase-1 levels, C-C motif chemokine 28 levels, and interleukin-5 levels, were associated with meningiomas. Further screening of inflammatory factors and positive MR analysis showed that FLT3 ligand levels had a clear causal association with the occurrence of meningioma (odds ratio [OR]=0.713, 95 % confidence interval [CI]: 0.598–0.851). The results of reverse MR analysis showed that there was a clear causal association between meningioma and Fms-related tyrosine kinase 3 ligand levels (OR=0.936, 95 % CI: 0.885–0.990). The results of heterogeneity and pleiotropic tests of MR-Egger intercept showed that there was no heterogeneity or pleiotropy in all data.

**Conclusions:**

This study clarified FLT3 as being involved in the pathogenesis of meningioma from a genetic perspective and genetically predicted lower FLT3L to be causally associated with a higher meningioma risk, implicating FLT3 signalling in meningioma pathogenesis. FLT3 as a genetically supported candidate factor associated with meningioma risk.

## Introduction

Meningioma originates from meningeal epithelial (arachnoid epithelial) cells. It is one of the common central nervous system tumours [1]. The incidence of meningioma increases with age [1], 2]. Meningioma risk factors include ionising radiation, obesity, and trauma [3], 4]. The World Health Organization (WHO) rates meningiomas according to 3 grades and 15 subtypes [5]. At present, for patients with small tumours and asymptomatic meningiomas, long-term observation of tumour growth can be used for conservative treatment. For patients with large tumours and symptoms, surgical resection is the preferred method; other treatments include radiotherapy and systemic treatment [3], 6]. For malignant meningiomas, the high recurrence and metastasis rates pose a significant challenge to the treatment of malignant meningiomas. The survival benefits for patients receiving traditional surgery, chemotherapy, and systemic treatment are not obvious [2].

The inflammatory tumour microenvironment, a systematic concept, comprises (among others) tumour cells and infiltrating inflammatory cells, stromal cells, chemokines, and cytokines. Inflammation, as the seventh feature of malignant tumours [7], has a complex effect on tumours. Inflammatory cells can release various cytokines and toxic mediators to exert their anti-tumour immune effects; however, inflammation also has a pro-tumour effect that can induce various pro-inflammatory cytokines, chemokines, and some proteases to be released into the tissue environment, causing oxidative damage, DNA mutation and microenvironment changes, thereby inducing the malignant transformation of cells and anti-tumour immunosuppression [8]. The emergence of cytokines and immune cells promotes the occurrence of inflammation, which leads to changes in the tumour microenvironment and the formation of a tumour-promoting inflammatory microenvironment. Changes in the tumour microenvironment can affect the metabolism of tissue, promote the malignant transformation of cells, and play a decisive role in the occurrence, survival, invasion, and metastasis of tumours [8], [9], [10]. The above studies have shown that the inflammatory tumour microenvironment is associated with the development and poor prognosis of a variety of tumours. To date, studies have explored the relationship between interleukin (IL)-6, IL-1β, tumour necrosis factor alpha (TNF-α) and meningiomas [11], but there are still many inflammatory factors and meningioma. The association between inflammatory factors and meningioma has not been reported.

Mendel’s randomisation (MR) research is an emerging epidemiological research method. Based on Mendel’s genetic law, MR uses genetic variation as an instrumental variable to explore the causal relationship between exposure and outcome. Therefore, it can skilfully overcome the influence of confounding factors generated in the acquired environment and avoid false negative or false positive results caused by the inevitable confounding factors and reverse causality in traditional observational studies. It is known as ’nature’s randomised controlled trial’ [12], 13].

At present, the causal relationship between many inflammatory factors and meningiomas is not known. This study uses the MR method to further explore the inflammatory factors related to meningioma to provide evidence for understanding the mechanism of the occurrence and development of meningiomas and formulating effective prevention and treatment strategies.

## Materials and methods

### Data source

In this study, 91 inflammatory factors were collected from relevant references [14]. The GWAS data number was (GCST90274758–GCST90274848), which can be downloaded from the EBI website (https://www.ebi.ac.uk/gwas/downloads/summary-statistics). The GWAS data of meningiomas were obtained from the FinnGen database (https://storage.googleapis.com/finngen-public-data-r10/summary_stats/finngen_R10_C3_MEN INGIOMA _ EXALLC.gz), including 1,316 cases and 313,392 controls. Most of the GWAS data in this study were derived from European populations. It should be noted, however, that the FinnGen database also includes a small proportion of individuals from non-European ancestries, although the exact proportion and specific ancestries are not detailed in the dataset documentation. To mitigate potential biases arising from population heterogeneity, we performed additional sensitivity analyses to assess the robustness of our findings. Specifically, we assessed the consistency of our results across different subgroups of the population. Although the FinnGen dataset primarily comprises European ancestries, we examined whether the inclusion of a small proportion of non-European individuals could influence our findings. The results of these sensitivity analyses indicated that our primary findings remained robust, with no significant deviations observed when considering potential population stratification.

### Filter instrumental variables

First, the Single Nucleotide Polymorphisms (SNPs) of each inflammatory factor were screened and summarised in turn. In this study, a series of strict criteria were used to select the instrumental variables (IVs) from the exposed GWAS summary data. The specific screening steps were as follows. 1) The SNPs were screened at a genome-wide significance level (p<5 × 10^−8^). 2) The SNPs (A/T or G/C) that were themselves palindromic sequences were excluded. The palindromic sequence of SNPs in the forward strand of DNA was the same as the base sequence on the reverse strand of DNA, and the direction was opposite. It was impossible to infer whether the chain was the forward strand or the reverse strand, so it was excluded. 3) Using r^2^<0.01 and a genetic distance of >10,000 kb as the value, the linkage disequilibrium (LD) in SNPs was excluded to screen for independent SNPs. Linkage disequilibrium was estimated according to the reference panel of the European population genome project. 4) To exclude the existence of weak instrumental variable bias, the F statistic of each SNP was calculated. The IVs with an F<10 were defined as weak instrumental variables and excluded in the MR analysis [15].

### Mendelian randomisation analysis process

First, MR analysis was performed on the inflammatory factor data that were defined in Section 1.2, and a hotspot MR analysis map was drawn. Then, the data of inflammatory factors related to the outcome were screened for MR analysis, and a forest map was drawn. Next, reverse MR analysis was performed on the selected inflammatory factors and outcome data.

1.3 Mendelian randomisation analysis was performed using the ‘Two Sample MR package’ in R 4.4.0 software (R Foundation for Statistical Computing, Vienna, Austria), and MR-PRESSO analysis was performed using the ‘MRPRESSO’ package. The specific steps were as follows. (1) The MR method: In this study, inverse variance weighting (IVW) [16], MR-Egger regression [17], the weighted median method [18], and simple and weighted model methods were used for analysis. If there were no horizontal pleiotropic effects among SNPs, IVW analysis was the main result [19]. (2) Statistical heterogeneity: A Cochran Q test was used to evaluate whether there was statistical heterogeneity among SNPs, and p≤0.05 indicated that there was statistical heterogeneity [20]. (3) Level pleiotropy: The intercept term of MR-Egger regression, the MR-Mendelian Randomisation Pleiotropy Residual Sum and Outlier (PRESSO) test, and a funnel plot were used to analyse the level pleiotropy of SNPs. If the intercept term was not statistically significant compared with 0, it indicated the absence of level pleiotropy in SNPs. The results of the MR-PRESSO test showed that p>0.05 indicated a lack of level pleiotropy in SNPs. The results of the funnel plot analysis showed that the left and right distribution of SNPs was basically symmetrical, suggesting that there was no significant horizontal SNP pleiotropy. (4) Sensitivity analysis: The effect of a single SNP on the results of the IVW analysis was evaluated by the leave-one-out method. If a single SNP was removed, the results of the IVW analysis did not change significantly, suggesting that the SNP had no significant effect on the results of the IVW analysis [21].

The number of SNPs retained and their strength: We re-examined the FLT3-ligand GWAS (ebi-a-GCST90274831, n˜330 k) after applying the standard QC pipeline described in the manuscript (PALINDROMIC exclusion, LD-clumping at r^2^<0.01/10,000 kb, MAF≥1 %, and F-statistic ≥10); 18 independent genome-wide significant SNPs (p<5 × 10^−8^) were kept as instruments for FLT3 ligand levels. The mean F-statistic was 47.8 (range 21–96), indicating that weak-instrument bias was unlikely (first-stage R^2^=3.7 %). Therefore, we did not perform alternative robust MR methods.

## Results

### Preliminary mendelian randomisation analysis results

**Figure 1:**
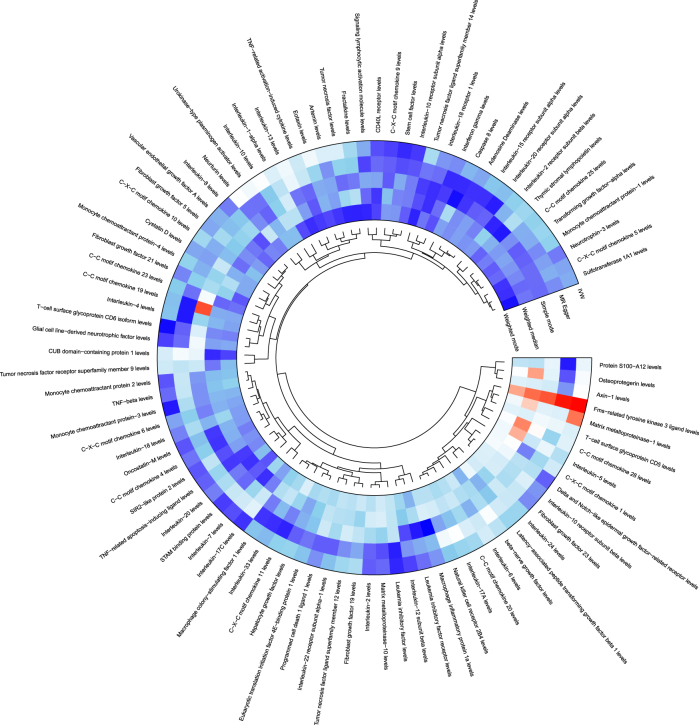
Mendelian randomization analysis results of 91 inflammatory factors.

After excluding weak instrumental variables, a total of 91 inflammatory factors were included in the preliminary MR analysis. The analysis results were summarised, and a heat map was drawn. The results showed that C-C motif chemokine 19 levels, osteoprotegerin levels, Fms-related tyrosine kinase 3 ligand levels, matrix metalloproteinase-1 levels, C-C motif chemokine 28 levels, and IL-5 levels were associated with meningiomas. See Figure 1 and Table 1.

**Table 1:** Preliminary MR analysis of six inflammatory factors was performed.

Name of inflammatory factor	Method	SNPs	*β*	SE	p-Value	OR (95 %CI)
Osteoprotegerin levels	MR Egger	27	−0.043	0.236	0.857	0.958 (0.603–1.521)
	Weighted median	27	−0.358	0.171	0.037	0.699 (0.500–0.978)
	IVW	27	−0.196	0.113	0.084	0.822 (0.659–1.027)
	Simple mode	27	−0.468	0.309	0.142	0.626 (0.342–1.148)
	Weighted mode	27	−0.334	0.186	0.084	0.716 (0.498–1.031)
Fms-related tyrosine kinase 3 ligand levels	MR Egger	45	−0.545	0.152	0.001	0.580 (0.430–0.781)
	Weighted median	45	−0.366	0.144	0.011	0.694 (0.523–0.920)
	IVW	45	−0.338	0.090	0.000	0.713 (0.598–0.851)
	Simple mode	45	−0.819	0.297	0.008	0.441 (0.246–0.789)
	Weighted mode	45	−0.331	0.148	0.030	0.718 (0.537–0.960)
C-C motif chemokine 28 levels	MR Egger	38	−0.191	0.247	0.445	0.826 (0.509–1.341)
	Weighted median	38	−0.365	0.166	0.028	0.694 (0.501–0.962)
	IVW	38	−0.155	0.114	0.175	0.856 (0.684–1.071)
	Simple mode	38	−0.623	0.307	0.050	0.536 (0.294–0.979)
	Weighted mode	38	−0.574	0.288	0.054	0.563 (0.320–0.990)
Matrix metalloproteinase-1 levels	MR Egger	26	0.279	0.208	0.192	1.322 (0.879–1.989)
	Weighted median	26	0.315	0.161	0.051	1.370 (0.999–1.879)
	IVW	26	0.269	0.111	0.015	1.309 (1.053–1.627)
	Simple mode	26	0.364	0.269	0.187	1.439 (0.850–2.436)
	Weighted mode	26	0.311	0.198	0.128	1.365 (0.927–2.012)
C-C motif chemokine 19 levels	MR Egger	36	−0.004	0.136	0.979	0.996 (0.764–1.300)
	Weighted median	36	−0.065	0.124	0.599	0.937 (0.735–1.194)
	IVW	36	−0.052	0.085	0.540	0.949 (0.804–1.121)
	Simple mode	36	−0.738	0.257	0.007	0.478 (0.289–0.791)
	Weighted mode	36	−0.063	0.113	0.581	0.939 (0.753–1.172)
Interleukin-5 levels	MR Egger	24	−0.426	0.279	0.141	0.653 (0.378–1.129)
	Weighted median	24	−0.379	0.165	0.022	0.685 (0.495–0.946)
	IVW	24	−0.136	0.144	0.344	0.873 (0.658–1.157)
	Simple mode	24	−0.369	0.316	0.255	0.691 (0.372–1.284)
	Weighted mode	24	−0.414	0.251	0.113	0.661 (0.404–1.081)

Among the 91 inflammatory factors included in our preliminary MR analysis, classical cytokines such as TNF-α and IL-1β were also screened. However, no significant causal associations were observed between these classical cytokines and meningiomas. This may have been due to several reasons. First, the genetic instruments available for these cytokines in the database may not be sufficiently powered to detect a causal effect. Second, the coverage of cytokine measurements in the database may be limited, which could influence the reliability of our conclusions regarding these specific factors. Future studies with more comprehensive genetic data and larger sample sizes are needed to further investigate the potential causal relationships between classical cytokines and meningiomas.

### Positive Mendelian randomisation analysis of disease-related inflammatory factors

**Figure 2:**
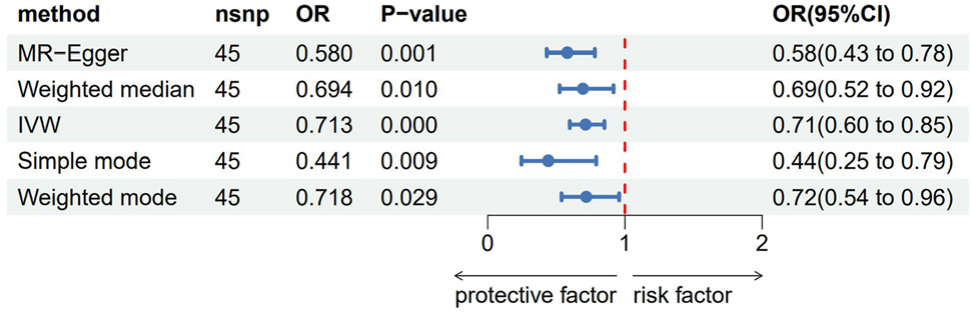
The analysis results of five methods of Fms-related tyrosine kinase 3 ligand levels MR. OR: Odds ratio, CI: Confidence interval.

Among the six associated inflammatory factors, one disease-related inflammatory factor (as shown in Table 2) was screened using the IVW method <0.05 and FDR <0.2, and MR analysis was performed on this inflammatory factor. The IVW results showed that Fms-related tyrosine kinase 3 ligand levels had a clear causal association with the occurrence of meningiomas (odds ratio [OR]=0.713, 95 % confidence interval [CI]: 0.598–0.851), as shown in Figure 2.

**Table 2:** Screening results of disease-related inflammatory factors.

Exposed ID	Name of inflammatory factor	SNPs	*β*	SE	IVW p-Value	OR (95 %CI)	FDR
GCST90274791	Fms-related tyrosine kinase 3 ligand levels	45	−0.338	0.090	0.000	0.713 (0.598–0.851)	0.015

### Reverse Mendelian randomisation analysis of disease-related inflammatory factors

**Figure 3:**
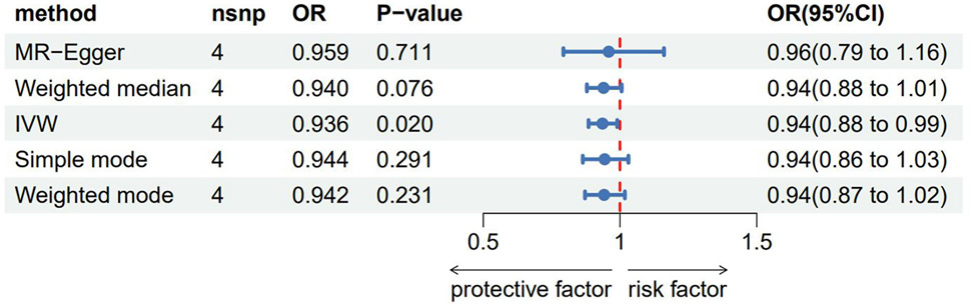
Reverse MR analysis of inflammatory factors and meningioma. OR: Odds ratio, CI: Confidence interval.

Reverse MR analysis was performed with meningiomas as the exposure factor and Fms-related tyrosine kinase 3 ligand levels as the outcome factor. The IVW analysis showed a clear causal association between meningiomas and Fms-related tyrosine kinase 3 ligand levels (OR 0.936, 95 % CI: 0.885–0.990), as shown in Figure 3. The results of the power calculation indicate that our study had a power of approximately 80 % to detect the observed effect size (OR=0.936) at the given significance level. This suggests that the observed marginal effect is robust and unlikely to have been the result of chance, given the large sample size and the moderate effect size. Although the reverse MR analysis yielded a nominally significant effect, this observation may reflect reverse causation or feedback bias rather than a true causal effect of meningiomas on FLT3 levels. Therefore, the claim of a two-way causal relationship should be tempered, and, accordingly, the biological mechanisms underlying the bidirectional association remain uncertain.

### Sensitivity analysis

**Figure 4:**
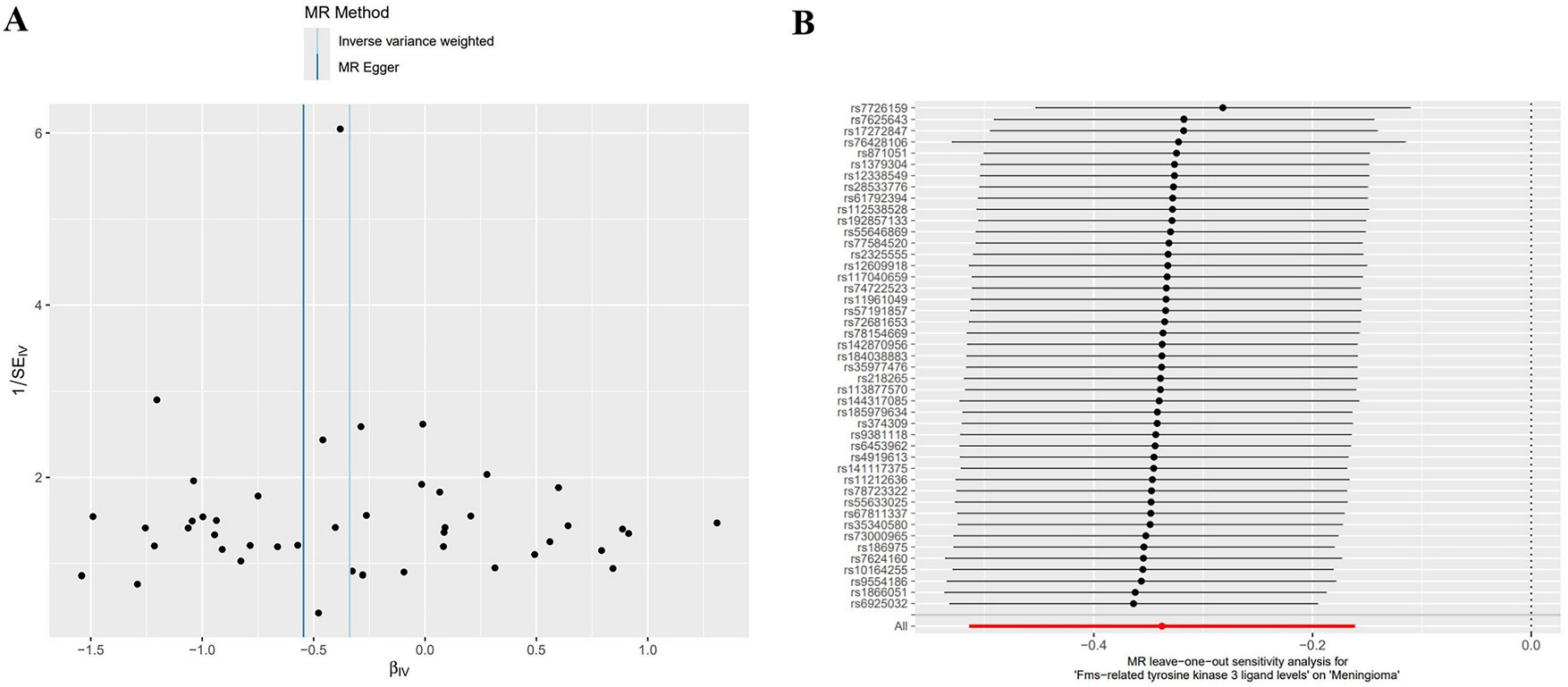
Positive MR sensitivity analysis, A: Funnel plot, B: Leave-one-out method.

In the forward MR analysis, the Cochran Q test results showed that there was no statistical heterogeneity among the SNPs highly correlated with Fms-related tyrosine kinase 3 ligand levels (Q=46.420, p=0.333). The results of the intercept analysis of the MR-Egger regression showed that there was no horizontal pleiotropy in SNPs that highly correlated with Fms-related tyrosine kinase 3 ligand levels (Egger intercept=0.027, p=0.101). The results of the funnel plot analysis showed that the left and right distribution of SNPs that highly correlated with Fms-related tyrosine kinase 3 ligand levels was essentially symmetrical, suggesting no significant level pleiotropy among SNPs highly correlated with Fms-related tyrosine kinase 3 ligand levels, as shown in Figure 4A. The results of the leave-one-out analysis showed that after removing a single SNP, the results of the MR analysis did not change significantly (see Figure 4B).

**Figure 5:**
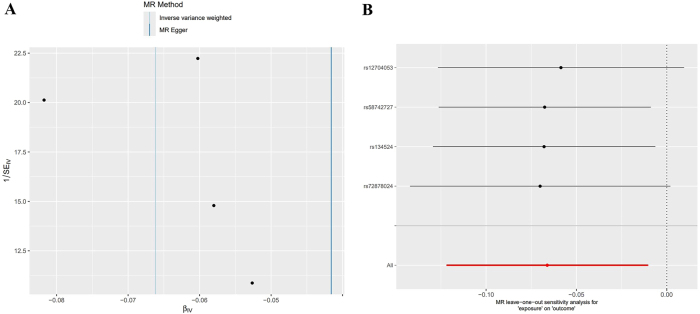
Reverse MR sensitivity analysis, A: Funnel plot, B: Leave-one-out method.

In the reverse MR analysis, the Cochran Q test results showed that there was no statistical heterogeneity between SNPs highly associated with meningiomas (Q=0.0821, p=0.960). The results of the intercept analysis of MR-Egger regression showed that there was no horizontal pleiotropy in SNPs that were highly correlated with meningiomas (Egger intercept=−0.008, p=0.817). The results of the funnel plot analysis showed that the left and right distribution of SNPs that were highly correlated with meningiomas was basically symmetrical, suggesting that there was no significant pleiotropic effect on the part of SNPs that were highly correlated with meningiomas, as shown in Figure 5A. The results of leave-one-out analysis showed that after removing a single SNP, the results of the MR analysis did not change significantly, as shown in Figure 5B.

## Discussion

Meningiomas are common intracranial tumours with a complex aetiology, and their development is influenced by various factors, including genetic and inflammatory factors [22], [23], [24]. Khan et al. [25] conducted a PRISMA-guided review of 127 studies, including laboratory and animal investigations, focused on pre-clinical modelling. The evaluation concluded that pre-clinical meningioma models provide valuable molecular insights into disease progression and effective chemo- and radio-therapeutic strategies for specific tumour subtypes. A large multicentre study conducted by Tosefsky et al. [26] presents longitudinal outcomes for grade 3 meningiomas with respect to recurrence, survival, and functional status. Their work confirms a survival benefit conferred by radiotherapy in this population and demonstrates that patients who remain alive five years after surgery maintain a favourable functional status. The results of this study show that Fms-related tyrosine kinase 3 (FLT3) is causally associated with meningioma. Fms-like tyrosine kinase 3 is a gene encoding tyrosine kinase, which can activate the cell proliferation pathway and promote the proliferation of hematopoietic stem cells [27]. The mutation, deletion, and overexpression of FLT3 can occur in acute myeloid leukaemia (AML). Warren et al. [28] found that FLT3 mutations were more common than FLT3 deletions, and almost all B-cell acute lymphoblastic leukaemia, 27 % of T-cell acute lymphoblastic leukaemia, and 89 % of AML have FLT3 overexpression, and patients affected by these have poor overall survival. In this study, in the forward MR, each 1-SD increase in FLT3-ligand level was associated with a 29 % reduction in meningioma risk (OR=0.713). The reverse MR indicated that a 1-unit increase in tumour liability lowered FLT3-ligand level by 6 % (OR=0.936). Although both effect sizes are modest, against a background lifetime risk of ∼0.9 % in the European population, an OR of 0.713 translates into an absolute incidence reduction of approximately 0.3 %. This preventive benefit is comparable to that accepted for aspirin in colorectal cancer (NNT˜300–400), suggesting the FLT3 signalling pathway as a potential target for chemoprevention in high-risk populations.

A proto-oncogene, Fms-related tyrosine kinase 3 is essential for the development of hematopoietic cells [29]. The importance of FLT3 gene mutation in solid tumours is not yet clear. Comprehensive analysis of FLT3 mutations found that somatic mutations accounted for the majority, followed by gene amplification [30]. Additionally, FLT3 amplification was found in solid tumours [27], where breast cancer accounted for 13.8 % [31], colorectal cancer accounted for 5.5 % [32], gastric cancer accounted for 1.7 % [33], and lung adenocarcinoma accounted for 0.4 % [34]. Lim et al. [27] found that FLT3 amplification was present in most gastrointestinal tumours. Studies have found that the mRNA levels of PDGFRA/B and FLT3 were significantly reduced after treatment with boccitinib in meningiomas and other solid tumours [35]. In addition, it has been reported that FLT3 is expressed in meningiomas, but its clinical significance in the context of meningiomas remains unclear [36].

In this study, the reverse MR analysis suggests that meningiomas affect FLT3-ligand levels. The FLT3-ligand (FLT3L) is normally produced by endothelial cells, fibroblasts, and activated T cells. Once a meningioma is established, it expresses FLT3 and may, therefore, internalise or ‘trap’ circulating FLT3L, lowering plasma levels [37]. Meningiomas are strongly infiltrated by lymphocytes and secrete TGF-β and IL-10 [38]. These cytokines down-regulate FLT3L transcription in stromal cells, providing a second route by which the tumour could suppress its own ligand [39]. Additionally, FLT3L expression is partly PI3K-dependent [40]. Meningiomas harbour constitutive PI3K/AKT activity and could, therefore, create a negative feedback loop that reduces ligand production [41].

According to existing research evidence, FLT3 is mainly expressed in bone marrow and lymphoid progenitor cells in patients with haematological malignancies. Receptors bind to FLT3 ligands and activate various downstream targets, including proteins in signal transduction and transcriptional activators (STAT), mitogen-activated protein kinase (MAPK) and protein kinase B (AKT) pathways, all of which are involved in regulating cell proliferation, differentiation, and cell survival [42]. Furthermore, FLT3 mutations are particularly common in AML, cases of myelodysplastic syndrome, and other haematological malignancies. In AML, FLT3 mutations account for about 30 % of all mutations [27], of which FLT3-ITD mutations account for 20–25 % [43], and FLT3-TKD mutations account for 5–10 % [28], 44]. There are few AML types with both FLT3-ITD and FLT3-D835 (the most common type of FLT3-TKD) mutations. Warren et al. [28] found FLT3 mutation to be unstable, and FLT3 mutation in patients with AML will change alongside the disease course. For example, AML without FLT3 mutation has mutation, on the one hand, because of the instability of FLT3 gene; on the other hand, the number of FLT3 mutations in the bone marrow of leukaemia cells at the time of initial diagnosis is very small and cannot be detected. The FLT3 mutations can cause abnormal activation of multiple downstream pathways, such as phosphatidylinositol 3-kinase (PI3K)/AKT, MAPK/extracellular signal-regulated kinase (ERK), and STAT5, leading to the uncontrolled proliferation of leukaemia cells [45]. In the context of meningiomas, FLT3 may activate several downstream signalling pathways that contribute to tumorigenesis. For instance, FLT3 can activate the PI3K/AKT pathway, which is known to promote cell survival and proliferation [45]. Additionally, FLT3 signalling may involve the MAPK/ERK pathway, which is implicated in cell differentiation and growth [45]. These pathways are commonly dysregulated in various cancers, including meningiomas, suggesting that FLT3 may drive meningioma progression through these mechanisms. Although FLT3 inhibition could potentially be used as a therapeutic strategy, future preclinical and clinical studies are needed to evaluate the potential benefits of FLT3 inhibition in meningioma treatment.

Cells in the blood are closely related to the tumour microenvironment; for example, tumour cells express a series of cytokines and chemokines during the course of the tumor disease, such as CXCL5, CXCL6, and CXCL8. Chemokine receptors CXCR1 and CXCR2, expressed by neutrophils, can combine with them and, in this way, be recruited to the tumour lesion area [46]. The proven CXCR2/CXCL5 axis promotes epithelial-mesenchymal transition, which, in turn, enhances the invasiveness of hepatocellular carcinoma [47]. In addition, the infiltration of T lymphocytes and B lymphocytes has been confirmed in most meningioma microenvironments, and Tregs play a major role in it. Under physiological conditions, TRegs control the body’s immune tolerance to antigens produced by itself; however, in a state where cancer is present, the number of TRegs is significantly increased, and the release of TGF-β and IL-10 further promotes the formation of an immunosuppressive environment and also limits the anti-tumour immunity of CD4+T lymphocytes, CD8+T lymphocytes, and natural killer cells [48]. In meningiomas, the infiltrating lymphocytes are mainly T lymphocytes. The anti-tumour immunosuppressive microenvironment mediated by TRegs has been confirmed in anaplastic meningiomas, which promotes the latter’s invasion ability [49]. At the same time, TRegs can break the link between antigen-presenting cells and tumour cells, weakening the anti-tumour effect of the immune system [50]. In conclusion, the above description is highly speculative, and the relationship between FLT3 and meningioma still needs further study.

Building on our MR-derived genetic evidence and contemporary neuro-oncological imaging standards, we now emphasise that future imaging–genomics models should integrate 5 complementary domains. (1) Intrinsic tumour features: volume, location, dural-tail length, enhancement pattern, quantitative ADC and DKI metrics, DCE-MRI kinetics (Ktrans, Ve), MRS metabolite ratios (Cho/Cr, Cho/NAA, Ala), and SWI/QSM micro-haemorrhage/calcification burden. (2) Peritumoral microenvironment markers: the extent of T2/FLAIR oedema, perfusion parameters from ASL-CBF and IVIM (f, D*), and radiomic texture entropy that correlates with CD68+ infiltrates. (3) Germline and circulating molecular data: genetically predicted FLT3L (this study), serum FLT3L protein, and additional inflammatory-protein QTLs to test the ‘low-FLT3L high-aggressiveness’ hypothesis. (4) Conventional clinical–pathology variables: age, sex, prior radiation treatment, WHO grade, Ki-67, and PR status. (5) Treatment-context factors: previous surgery or radiosurgery margins that modulate post-treatment imaging appearances. Collectively, these multi-level inputs will maximise the sensitivity and specificity of imaging-based risk stratification and help translate our genetic findings into clinically actionable imaging biomarkers.

This study also has some limitations. First, the GWAS data related only to the European population and, as such, the results may not be applicable to other populations. Further research that includes a diverse range of populations is needed. Second, although we found a causal relationship between FLT3 and meningioma, the exact biological mechanisms remain unclear. More studies are needed to explore how FLT3 affects meningioma development. Although FLT3 is known to activate PI3K/AKT and MAPK/ERK signalling in hematologic malignancies, current support for these pathways in the presence of meningiomas is indirect and extrapolated from other cancers. Dedicated functional studies (e.g. FLT3 knock-down/knock-in experiments in primary meningioma cultures or animal models) are required to validate whether these cascades mediate the observed effect. Third, the roles of other potential genetic factors identified in this study are not fully understood. Population studies and gene-protein association studies could help to clarify their involvement in meningioma. Finally, despite our efforts to assess pleiotropy, we cannot completely rule out its potential impact on the results. Further research with more advanced methods is needed to address this issue. Although this study focused on the causal link between inflammatory markers and meningioma risk, their potential role in predicting peritumoral oedema outcomes remains unexplored and represents an important area for future investigation.

## Conclusions

In this exploratory two-sample MR analysis of European-ancestry GWAS data, we identified FLT3 as a genetically supported candidate factor associated with meningioma risk. These findings should be viewed as hypothesis-generating, rather than conclusive evidence of a therapeutic target. We strongly recommend independent replication in multi-ancestry cohorts and experimental studies to clarify the biological mechanisms linking FLT3 to meningioma development before any clinical translation is contemplated.
